# The Diagnostic Dilemma of Malignant Biliary Strictures

**DOI:** 10.3390/diagnostics10050337

**Published:** 2020-05-25

**Authors:** Robert Dorrell, Swati Pawa, Yi Zhou, Neeraj Lalwani, Rishi Pawa

**Affiliations:** 1 Department of Medicine, Wake Forest School of Medicine, Winston-Salem, NC 27157, USA; rdorrell@wakehealth.edu; 2 Division of Gastroenterology, Department of Medicine, Wake Forest School of Medicine, Winston-Salem, NC 27157, USA; spawa@wakehealth.edu; 3 Department of Pathology, Wake Forest School of Medicine, Winston-Salem, NC 27157, USA; yozho@wakehealth.edu; 4 Department of Radiology, Wake Forest School of Medicine, Winston-Salem, NC 27157, USA; nlalwani@wakehealth.edu

**Keywords:** malignant biliary stricture, hepatobiliary malignancy, pancreatic cancer, endoscopic ultrasound, fine-needle aspiration and biopsy, endoscopic retrograde cholangiopancreatography, cholangioscopy, intraductal ultrasound, confocal laser endomicroscopy

## Abstract

The differential diagnosis for biliary strictures is broad. However, the likelihood of malignancy is high. Determining the etiology of a biliary stricture requires a comprehensive physical exam, laboratory evaluation, imaging, and ultimately tissue acquisition. Even then, definitive diagnosis is elusive, and many strictures remain indeterminant in origin. This literary review examines the diagnostic dilemma of biliary strictures and presents innovations in both histochemical and endoscopic techniques that have increased the diagnostic power of differentiating benign and malignant strictures. The field of tissue biopsy is revolutionizing with the advent of free DNA mutation profiling, fluorescence in situ hybridization (FISH), and methionyl t-RNA synthetase 1 (MARS 1), which allow for greater testing sensitivity. Endoscopic ultrasound, endoscopic retrograde cholangiopancreatography (ERCP), cholangioscopy, confocal laser endomicroscopy, and intraductal ultrasound build upon existing endoscopic technology to better characterize strictures that would otherwise be indeterminate in etiology. This review uses recent literature to insert innovative technology into the traditional framework of diagnostic methods for malignant biliary strictures.

## 1. Introduction

A biliary stricture is a narrowing of the biliary tree that can be caused by a myriad of etiologies, some benign, some life-threatening. There are three classes of biliary strictures: benign, malignant, and indeterminate. Unfortunately, only a minority of biliary strictures (15%–24%) are benign [1]. Differentiating between these benign and malignant strictures requires a complex diagnostic evaluation. Endoscopy is often vital to diagnosis through tissue sampling. However, recent advances in understanding and utilizing biomarkers are enhancing the diagnostic power of laboratory testing. This literary review attempts to present the diagnostic dilemma of identifying a stricture as malignant.

## 2. Etiology

The most common cause of malignant stricture of the distal common bile duct is pancreatic adenocarcinoma. This occurs when the pancreatic tumor invades the common bile duct. Because pancreatic cancer is often diagnosed at a later stage, 70% of patients with pancreatic cancer already have a biliary stricture at the time of diagnosis [2,3]. The second most common cause of malignant biliary stricture is cholangiocarcinoma, a primary tumor of the bile duct itself. A minority of cases are caused by other etiologies including primary duodenal adenocarcinoma, ampullary carcinoma, gallbladder carcinoma, hepatocellular carcinoma, lymphoproliferative disorders, and metastatic lesions [4]. Malignancies of the hepatopancreatic biliary (HPB) system are largely sporadic, but certain inflammatory conditions like primary sclerosing cholangitis, recurrent or chronic infections like cholangitis or pancreatitis, and even cholelithiasis are risk factors for developing a malignancy [5]. All biliary strictures should be taken seriously and evaluated thoroughly given the high chance of malignancy.

## 3. Presentation and Laboratory Markers

The initial evaluation of biliary strictures includes physical exam and laboratory markers. Patients often present with malaise, weight loss, anorexia, jaundice, pruritis, nausea, and vomiting. These symptoms are generally associated with hyperbilirubinemia, which occurs due to the stricture’s blockage of bile excretion from the gallbladder to the small intestine [6]. As bilirubin levels rise, the symptoms typically progress surreptitiously until they have a major impact on the patient’s quality of life. More advanced obstructions can cause more fulminant symptoms secondary to infections like ascending cholangitis or hepatic abscesses [6]. Patients presenting with any of the symptoms over this wide spectrum should be examined for icterus as well as hepatosplenomegaly and lymphadenopathy. In turn, laboratory tests should include bilirubin levels as well as other markers of hepatobiliary dysfunction including aspartate aminotransferase (AST), alanine aminotransferase (ALT), alkaline phosphatase (ALP), and gamma-glutamyl transferase (GGT). The greater the bilirubin level, the more likely that the stricture is malignant [7]. The abnormal liver biochemistry also follows the typical obstructive pattern, with ALP rising more than AST [8]. Furthermore, Thomasset et al. studied the relationship between initial laboratory results and the ultimate diagnosis of biliary stricture etiology. Based on their assessment of 830 patients with presumed biliary strictures, normal liver function tests (LFTs) help to rule out primary HPB malignancies. However, abnormal LFTs, even in the presence of normal bilirubin levels, were associated with a higher likelihood of malignant stricture. Therefore, isolated or combined abnormalities of bilirubin and LFTs confer a greater risk that a biliary stricture is malignant [9].

## 4. Biomarkers

While standard laboratory tests can be somewhat helpful in determining etiology, more specific tests like biomarkers give better insight into the absence or presence of HPB malignancy causing stricture. The most commonly used tumor marker in this setting is cancer antigen 19-9 (CA 19-9), which is a carbohydrate antigen expressed on the surface of certain cancer cells [10,11]. The antigen sheds from these cell surfaces attached to various carrier proteins and can be detected in the bloodstream. CA 19-9 is typically associated with pancreatic cancer, but it can also be elevated with cholangiocarcinoma, cholestasis, cholangitis, cirrhosis, pancreatitis, and any cause of biliary obstruction [11,12,13]. Therefore, this marker cannot be used to reliably determine the etiology of a biliary stricture.

Recent CA 19-9 research has focused on using the biomarker to better differentiate between malignant and benign processes of biliary obstruction. For example, Yue et al. measured CA 19-9 in conjunction with certain carrier proteins. The carrier proteins seem to be more specific to the organ of origin and can help to focus the differential of elevated CA 19-9 [12]. Other studies have shown that CA 19-9 levels are higher in malignant processes compared to benign. Therefore, a higher cutoff value for CA 19-9 decreases sensitivity, but increases specificity for malignant processes [14,15,16]. La Greca et al. also proposed correcting CA 19-9 levels for the presence of biliary obstruction and inflammation by calculating the CA 19-9 to total bilirubin ratio and CA 19-9 to CRP (C reactive protein) ratio, respectively. This study found that using ratios compared to CA 19-9 alone decreased the sensitivity, but improved the specificity for malignant biliary obstruction [15]. While Liu et al. only found a mild improvement in specificity using the CA 19-9/bilirubin ratio (83%) compared to CA 19-9 levels alone (81%), this study also reported on the effects of CA 19-9 levels plus the CA 19-9/bilirubin ratio. The combination of the two values compared to CA 19-9 alone decreased sensitivity to 62%, but increased specificity from 81% to 93% and increased diagnostic accuracy from 74% to 81% [17]. As the scope of CA 19-9 testing broadens and is better understood in the context of biliary obstruction and inflammation, this tumor marker may become a more reliable indicator of HPB malignancies in the future.

Carcinoembryonic antigen (CEA) is similar to CA 19-9, as it is a tumor marker that can be associated with a wide range of pathologies. However, the utilization of CEA at present is even less given its low sensitivity (30%–68%) and specificity (75%–95%) for cholangiocarcinoma [12].

## 5. Non-Invasive Imaging Studies

**Figure 1:**
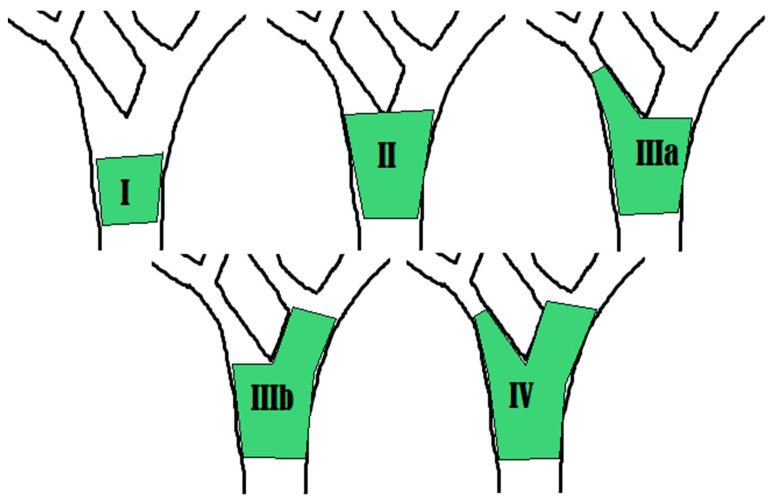
The Bismuth–Corlette classification is a system for characterizing hilar strictures. Type I is limited to the common hepatic duct, below the main confluence of the hepatic ducts. Type II involves the confluence of the left and right hepatic ducts. Type IIIa involves the main hepatic confluence and extends to the bifurcation of the right hepatic duct. Type IIIb involves the main hepatic confluence and extends to the bifurcation of the left hepatic duct. Type IV involves the main, right, and left hepatic confluence.

Imaging studies play an essential role in the visualization, classification, and surgical planning of biliary strictures. The goal of imaging is to first assess for dilation of the intrahepatic and extrahepatic biliary tree. Different modalities have established different cutoff values for pathologic dilation of the common bile duct (CBD) and intrahepatic bile ducts. The second goal is to pinpoint the level of obstruction. This location is typically described using the Bismuth–Corlette classification system (Figure 1), which groups biliary strictures into four different types depending on their location along the biliary tree. Ancillary imaging findings include characteristics of the duct walls like thickness and texture [6,18]. Imaging can be broken into two categories: non-invasive and invasive. In the algorithm of evaluating a malignant biliary stricture, non-invasive imaging typically precedes invasive imaging.

Non-invasive imaging modalities of biliary strictures include right upper quadrant abdominal ultrasound (RUQUS), computed tomography (CT), contrast-enhanced magnetic resonance imaging (MRI) and magnetic resonance cholangiopancreatography (MRCP). RUQUS visualizes the liver, gallbladder, biliary tract, and pancreas and is often the first tool providers reach for when a patient presents with symptoms of obstructive jaundice. The benefits of RUQUS include low cost, lack of radiation, and high sensitivity to detect biliary dilation or obstruction, with an accuracy of more than 90%. The limitations of this imaging modality include poor visualization of strictures in obese patients and low accuracy in identifying the etiology of a biliary obstruction (30–70%) [19,20].

**Figure 2:**
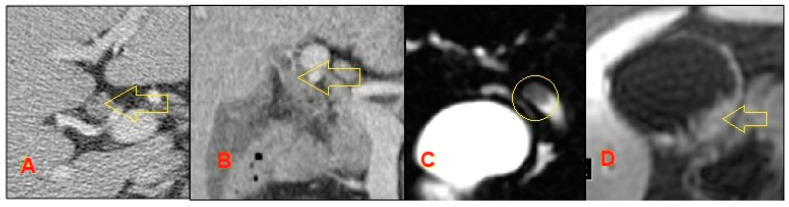
**A**,**B**: Axial contrast computed tomography (CT) shows enhancing intraductal polypoid mass consistent with intraductal cholangiocarcinoma (arrow); **C**: The circle demonstrates a T2W hypointense lesion found on magnetic resonance cholangiopancreatography (MRCP); **D**: Postcontrast MR showing an incidentally found subtle mural thickening of the gallbladder fundus later diagnosed as synchronous gallbladder carcinoma.

Compared to RUQUS, MRCP with contrast-enhanced MRI is not as limited by body habitus, obtains a more detailed view of the biliary system, and captures extra-biliary structures to give a broader sense of the stricture in context [21,22]. Its images are detailed enough to determine the level of biliary obstruction, with 98% sensitivity and specificity. MRCP with contrast-enhanced MRI may also differentiate between benign and malignant strictures, with a sensitivity of 38%–90% and a specificity of 70%–85%. It can also help in the staging of cholangiocarcinomas and determining surgical management [23]. MRCP is preferred over its invasive counterpart endoscopic retrograde cholangiopancreatography (ERCP) for initial evaluation of biliary strictures due to fewer side effects and a similar ability to visualize the stricture [22,23,24] (Figure 2).

**Figure 3:**
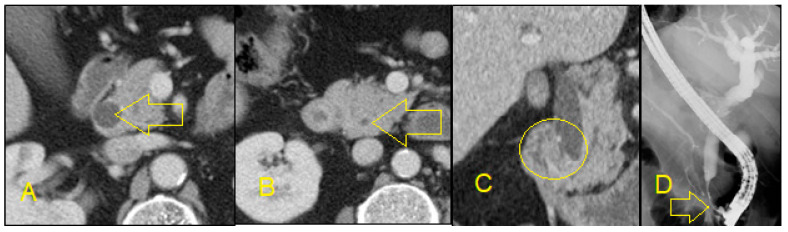
**A**: Axial contrast-enhanced CT shows enhancing dilated common duct (arrow); **B**: there is abrupt cutoff of the common duct and there is diffuse mural thickening and enhancement (arrow); **C**: The circle demonstrates an infiltrative mass in the distal duct found on coronal CT; **D**: endoscopic retrograde cholangiopancreatography (ERCP) shows a short-segment tight stricture corresponding to the mass. Biopsy confirmed cholangiocarcinoma.

**Figure 4:**
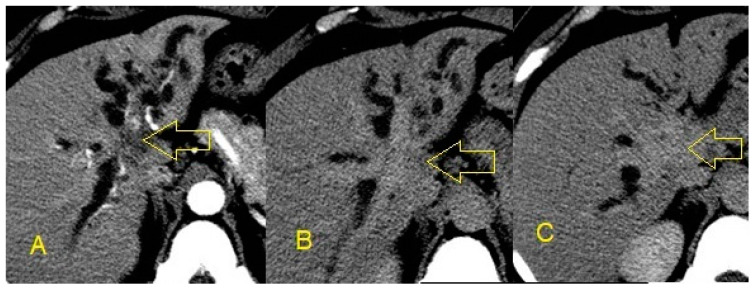
**A**: Axial arterial phase CT shows dilated intrahepatic ducts and a hypodense mass (arrow) at the hilum; **B,C**: venous phase images show enhancement of the mass. This signifies excessive fibrous stroma in the tumor and is consistent with cholangiocarcinoma.

**Figure 5:**
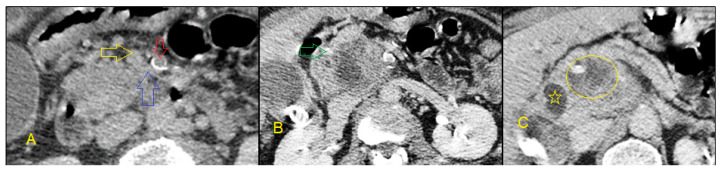
**A**–**C**: CT shows a pancreatic head mass with central necrosis (circle, C). Additional findings include no calcifications to support underlying chronic pancreatitis, no ductal dilatation, peripancreatic fat planes are not maintained (blue arrow, A) and superior mesenteric artery (SMA) (red arrow, A)/superior mesenteric vein (SMV) (yellow arrow, A) ratio is ≥ 1. The gastroduodenal artery (green arrow, B) is encased by the mass. Dilated common bile duct (star, C). Overall, there are at least three signs suggesting the diagnosis of malignancy.

Contrast-enhanced CT scans are useful for identifying HPB masses and delineating the extent of masses by showing tissue and vessel infiltration (Figure 3). For this reason, they are helpful in the initial diagnosis of a mass as well as determining surgical resectability and planning surgical interventions [25]. A special CT protocol with delayed (20 min) images is particularly valuable in suspected cases of cholangiocarcinoma (Figure 4 and Figure 5). In summary, non-invasive imaging modalities are useful to determine presence and location of stricture, but invasive imaging is needed to obtain a diagnosis.

## 6. Invasive Imaging Methods

Invasive imaging methods are required to obtain tissue samples from biliary strictures and make a definitive diagnosis. Invasive methods are also able to directly visualize biliary obstruction; however, these modalities are more likely to have complications than non-invasive methods. The field of invasive imaging is rapidly expanding past the more traditional ERCP, endoscopic ultrasound–fine-needle aspiration (EUS–FNA), and percutaneous transhepatic cholangiography (PTHC) to include cholangioscopy, intraductal ultrasound, and confocal laser endomicroscopy.

### 6.1. Endoscopic Retrograde Cholangiopancreatography

ERCP-guided tissue acquisition is a useful modality in patients presenting with obstructive jaundice secondary to biliary stricture requiring drainage. The procedure involves passage of a duodenoscope through the mouth and into the duodenum. From the duodenum, a catheter is threaded through the ampulla into the common bile duct over a guidewire. Following cannulation of the bile duct, contrast is injected through the cannula for fluoroscopic imaging and better delineation of the biliary tree. Because of its ability to obtain tissue and alleviate obstruction, ERCP is the preferred method for patients with obstructive jaundice and systemic symptoms in whom a diagnosis cannot be achieved with EUS–FNA alone [26].

**Figure 6:**
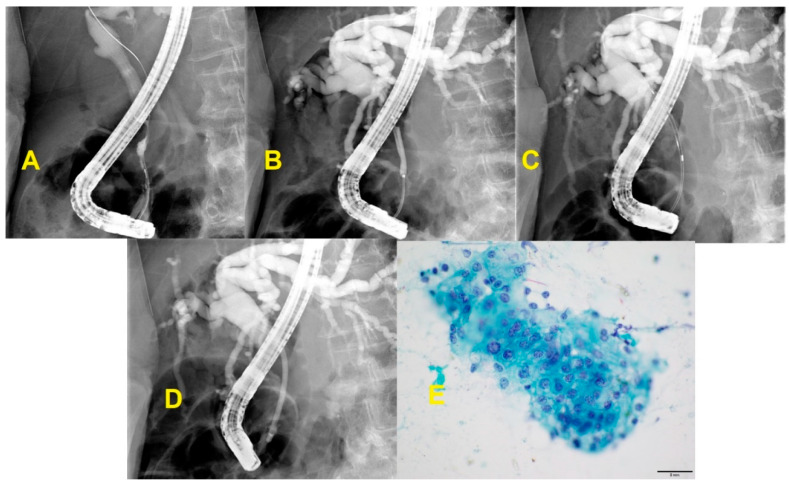
ERCP with brushings and resultant cytology. **A**: Cholangiography showing distal bile duct stricture with upstream ductal dilatation. **B**: Balloon dilation of distal bile duct stricture. **C**: Brushings obtained from distal bile duct stricture during ERCP. **D**: Plastic stent placed in bile duct across stricture for biliary drainage. **E**: Brush cytology showed biliary tract adenocarcinoma. The group shows loss of polarity, irregularly spaced nuclei. The nuclei are angulated and pointed with subtle grooves and folding. Small nucleoli are present in the cells (Papanicolaou stain).

There are two methods of obtaining tissue during ERCP: brushings of the biliary stricture for cytologic evaluation and forceps for intraductal biopsies under fluoroscopic guidance. While ERCP has high specificity (95%) for diagnosing malignancy within biliary strictures, its sensitivity is low. Biliary brushings have a sensitivity of 23%–56%, biopsies have a sensitivity of 33%–65%, and the combination of the two has a sensitivity of 60%–70% [23]. Roth et al. demonstrated that cytology of both the brushings and biliary fluid, aspirated from the biliary tree before and after brushings, increased sensitivity to 84%. A newer method of obtaining tissue through ERCP is scraping. Rather than using a brush to obtain a sample from the stricture, the Trefle Biliary Scraper uses a looped metal wire to shave cells from a biliary stricture [27]. Nakahara et al. showed that the sensitivity of this method for diagnosing malignancy was 41%, which was significantly more sensitive than traditional cytology [28]. However, even the scraper’s sensitivity is low, and many malignancies will be missed using these ERCP techniques. (Figure 6)

More advanced cytological techniques have been developed to improve sensitivity of brush cytology. Obtaining cells from the brushes once they are removed from the body involves either smearing the brush directly onto a glass slide or placing the brush in a preservative fluid and then centrifuging this liquid to isolate the cells. Either way, the samples are fixed and stained with various stains, including Papanicolaou and May-Grünwald Giemsa. Pathologists can then identify pathologic or malignant cell features through microscopic evaluation [25]. Researchers have recently expanded upon conventional brush cytology to identify other markers of malignancy within the brushed cells, including microRNA (miRNA), transfer RNA (tRNA), and DNA mutations. miRNA has proved an important biomarker for diagnosis of biliary malignancies. This molecule also enhances sensitivity of brush cytology. Le et al. showed that the sensitivity of brush cytology increased from 54% to 85% when adding miRNA staining [29]. Jang et al. recently studied staining biliary samples for methionyl-tRNA synthetase 1 (MARS1). tRNA molecules are integral to protein catalysis within cells and they also play a role in cancer development. Immunohistochemical and immunofluorescent staining for MARS1 had a sensitivity of 70% and specificity of 96% for diagnosing malignancy for 80 patients with biliary strictures [30]. Fluorescence in situ hybridization (FISH) has also been studied on brush cytology specimens. Because FISH testing identifies chromosomal abnormalities in 80% of biliary cancers, researchers hypothesized that FISH would improve identification of malignant biliary strictures from ERCP sampling. Indeed, FISH in combination with brush cytology did modestly improve sensitivity to 50%–60% [31].

Despite these new cytological markers, one barrier to high sensitivity with cytology is insufficient cellular sampling. Brush cytology may return inadequate cellularity due to factors like tissue fibrosis, tissue ulceration, and patterns of invasion such as submucosal spread [32]. Mutation profiling (MP) is advantageous because it samples free DNA rather than intact cells. The free DNA can be found in the preservative fluid holding the biliary brush after it is centrifuged and cells are removed. The DNA can then be evaluated for several mutations known to be associated with malignancy. Kushnir et al. reported that brush cytology plus MP had a sensitivity of 56%, which is significantly more sensitive than brush cytology alone. This article found that the highest sensitivity (66%–69%) was achieved by using brush cytology, FISH, and MP together. There was no difference in specificity amongst these three tests. Perhaps the greatest benefit of MP was increasing the diagnostic yield on brush samples from 22% for traditional cytology to 100% [27]. As the analysis of ERCP samples continue to be studied and expanded, ERCP will become even more adept at diagnosing malignant biliary strictures [31].

### 6.2. Cholangioscopy

Per Oral Cholangioscopy (POCS) involves passage of a choledochoscope through the working channel of a duodenoscope into the biliary tract. This provides direct, not just fluoroscopic, visualization of the biliary tract [4]. The recent introduction of a single operator digital cholangioscope called Spyglass DS (Boston Scientific Corp) has allowed POCS to become one of the primary tools in diagnosis of MBS [33].

**Figure 7:**
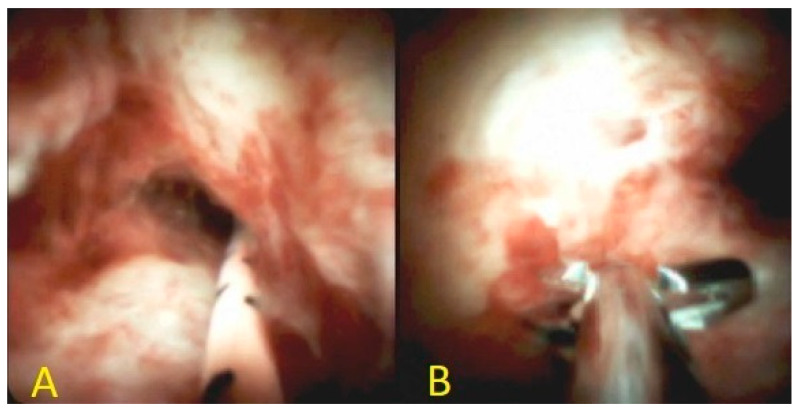
**A:** Cholangioscopy revealing hilar stricture with irregular mucosa and neovascularization. **B**: Stricture sampling with SpyBite biopsy forceps.

POCS allows for evaluation of the stricture based on its appearance as aberrant mucosal and vascular patterns are suspicious for malignancy. The visual findings of POCS alone have a sensitivity of 88.9% and specificity of 97.6% for predicting malignancy [34]. POCS also permits targeted tissue biopsies with a sensitivity of 71%–100% and a specificity of 96.7%–100% [33]. Additionally, POCS-directed biopsies have shown greater diagnostic ability than brushings and fluoroscopic-guided biopsies of biliary strictures obtained during ERCP [35] (Figure 7).

One of the barriers to widespread use of POCS is limited interobserver agreement (IOA). Sethi et al. reported an IOA of 45% [36]. This led Sethi et al. to develop the Monaco classification system to streamline IOA. They identified eight criteria including presence of stricture, presence of lesion, mucosal features, papillary projections, ulceration, abnormal vessels, scarring, and pronounced pit pattern. The adoption of this classification system increased IOA to 70% [37].

POCS should be utilized after EUS–FNA/FNB and standard ERCP techniques have failed to provide a diagnosis due to high cost and increased risk of adverse events with POCS [38]. In a large retrospective review of almost 4000 procedures, POCS showed an increased adverse event rate of 7% versus 2.9% with traditional ERCP [39]. Cholangioscopy is a promising technology; however, additional research is needed to standardize findings, increase IOA, and to reduce the complication rate [37].

### 6.3. Endoscopic Ultrasound-Guided Fine-Needle Aspiration

**Figure 8:**
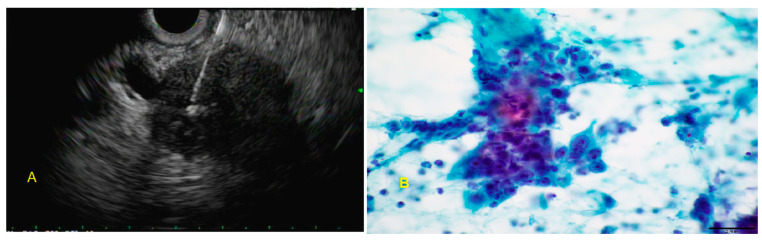
**A:** Endoscopic ultrasound (EUS)-guided fine-needle aspiration of a pancreatic head mass. **B**: The malignant cells are crowded and overlapping. The nuclei are enlarged and show nuclear size variation in a range of 1:3. (FNA, Papanicolaou stain).

Biliary strictures associated with a mass lesion found on cross-sectional abdominal imaging are often further imaged and sampled with endoscopic ultrasound-guided fine-needle aspiration (EUS–FNA). These mass lesions include primary HPB malignancy, metastatic tumors, and lymphoproliferative disorders. During this procedure, an echoendoscope is advanced into the upper gastrointestinal tract, where adjacent organs can be visualized with greater detail in comparison to non-invasive imaging. A FNA needle is advanced through the working channel and directed under real-time sonographic imaging toward the target lesion (Figure 8).

A fine-needle aspirate is obtained and cytological analysis is performed from this aspirate. This procedure has a sensitivity of 80% and a specificity of 97% to identify malignancy [31,40]. The benefits of EUS–FNA include not only high sensitivity, but also widespread availability, low cost, and the ability to evaluate lesions not seen on imaging [41]. EUS–FNA is favored over ERCP in asymptomatic patients who do not require biliary drainage [26]. Of note, EUS–FNA is typically avoided in hilar cholangiocarcinoma due to concern for tumor seeding [42]. This concern is the greatest barrier to widespread use of EUS–FNA. Yamaguchi et al. reported a case of a solid pseudopapillary neoplasm of the pancreas that was seeded into the gastric wall 5 years after the original EUS–FNA procedure [43]. Ultimately however, only three such cases have been reported in the literature. Therefore, concern for malignant peritoneal seeding should not preclude utilization and further investigation of this effective diagnostic tool [44].

### 6.4. Endoscopic Ultrasound-Guided Fine-Needle Biopsy

**Figure 9:**
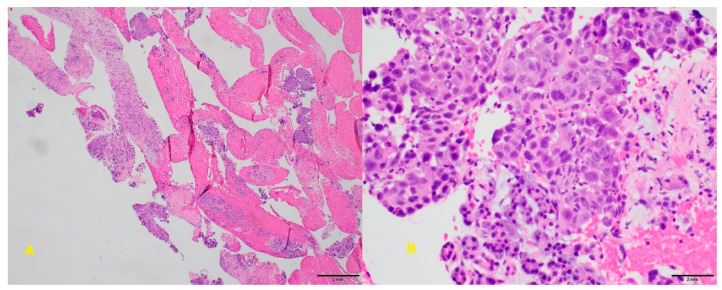
**A**: Malignant glands admixed with normal pancreatic parenchyma (core biopsy, H&E stain). **B**: On high power, the tumor cells form solid nest with focal glandular formation. There is nuclear enlargement and irregularity, abnormal chromatin, and prominent nucleoli (core biopsy, H&E stain).

Endoscopic ultrasound-guided fine-needle biopsy (EUS-FNB) is similar to EUS–FNA, but it uses a larger bore needle capable of obtaining core biopsies from the suspect lesion (Figure 9). Numerous studies have compared the efficacy of EUS-FNB to EUS–FNA. Van Riet et al. performed a randomized study of over 600 patients with biliary strictures. The FNB needle provided higher histologic yield (77% vs. 44%) and increased diagnostic accuracy (87% vs. 78%) compared to FNA [45,46]. EUS-FNB has also been shown to provide more core tissue and nucleic acid yield than FNA samples [47]. More studies are needed to demonstrate diagnostic superiority of FNB over FNA, especially when using rapid on-site evaluation (ROSE) [48]. ROSE involves immediate evaluation of the sample at the time of biopsy, thus determining whether the tissue is adequate for diagnosis. The diagnostic yield of EUS–FNA with ROSE was not significantly different than EUS-FNB without ROSE [49]. Overall, EUS-FNB provides a reliable alternative to EUS–FNA with increased diagnostic accuracy, especially when ROSE is not available.

Classically, only biopsies obtained during surgical procedures provided sufficient samples of the tissue in question. However, endoscopic samples are now adequate for genomic analysis and next generation sequencing (NGS) thanks to these improved techniques in endoscopic ultrasound tissue acquisition. Valero et al. assessed the variability between endoscopic biopsy and surgical biopsy with NGS and reported 100% endoscopic sample agreement with genomic sampling of surgically acquired specimens [50]. EUS-FNB microcore samples have better tissue integrity and microscopic tissue architecture over EUS–FNA, allowing for the use of innovative biomarker evaluation [51].

### 6.5. Intraductal Ultrasound

Intraductal ultrasound (IDUS) is a technique used during ERCP, in which an ultrasound probe is advanced through the working channel of the duodenoscope into the biliary system. IDUS provides real-time imaging of the stricture using high frequency ultrasound and helps to identify malignant characteristics including hyperechoic, asymmetric wall thickening, irregular borders, and abrupt shoulders. Studies have shown a sensitivity and specificity of 98% for diagnosis of MBS. While IDUS has an impressive sensitivity based on sonographic appearance of stricture alone, this technology can also be used for ultrasound-guided biopsies. In fact, IDUS-guided biopsy has demonstrated an improved sensitivity over fluoroscopic biopsy (87% vs. 67%). This technology is promising, but its role within the diagnostic algorithm of biliary strictures is still being refined [4,52,53,54].

### 6.6. Confocal Laser Endomicroscopy

Confocal laser endomicroscopy (CLE) is a novel endoscopic technology used to obtain real-time histopathologic diagnosis of biliary strictures. During this procedure, confocal miniprobes are passed through the ERCP catheter and fluorescein dye is administered to produce high-resolution images of the biliary stricture at a microscopic level. CLE probes visualize biliary epithelium by transmitting low-power laser and detecting light reflected back from the tissue. Almadi et al. studied CLE effectiveness in evaluating biliary strictures in comparison to ERCP. They noted an increased sensitivity with a combination of CLE and ERCP (98%) in comparison to ERCP alone (45%) [55,56]. However, specificity and the positive predictive value were decreased with the combination. As a result, they concluded that CLE should be reserved for strictures that are still indeterminate after intraductal ultrasound and cholangioscopy [57].

CLE requires additional training and does not provide a significant advantage over other technologies. Provider variability is considered CLE’s greatest obstacle to widespread use. The Miami classification was developed to streamline and advance its accuracy over other technologies. This classification system describes four criteria that are considered highly suspicious for malignancy. These include thick white bands, thick dark bands, dark clumps, and epithelial structures. The presence of one feature renders a 97% sensitivity and 33% specificity for identifying malignancy [58,59]. Due to Miami’s low specificity, the Paris classification was developed to describe benign biliary strictures. Benign strictures are characterized by thickened reticular strictures, multiple thin white bands, increased spaces between scales, and dark granular patterns with scales. Additional studies are needed to determine whether combining Miami and Paris classifications will improve diagnostic utility of CLE. Until further research is conducted, CLE should remain reserved for indeterminate strictures after other advanced invasive diagnostic techniques are utilized [4]. Sensitivity and specificity of various MBS diagnostic modalities can be found in Table 1.

### 6.7. Percutaneous Transhepatic Cholangiography

PTHC is a radiologic procedure that directly accesses the biliary system through ultrasound-guided percutaneous needle placement into the bile duct. This allows for fluoroscopic imaging of the biliary tree with injected contrast, biopsies of biliary tissue, drainage of fluid upstream of the obstruction, and cytology on the drained fluid to aid in diagnosing stricture etiology [60]. PTHC is reserved for patients when diagnosis is not achieved with invasive imaging or when patients are too unstable to undergo endoscopic procedures [61]. This procedure is only used for such select cases because it has a complication rate ranging from 0.5% to 2.5% [62].

Overall, endoscopic procedures are quickly evolving to increase diagnostic power for diagnosing malignant biliary strictures. These procedures require additional training for providers, and are associated with high risk for complications, particularly cholangitis [63]. However, as these techniques are refined and further studied, they will likely streamline the diagnosis of malignant biliary strictures, with fewer procedures required to make a definitive diagnosis.

## 7. Conclusions

Prompt diagnosis of MBS with biomarkers, contrast-enhanced CT, MRCP with contrast-enhanced MRI, and advanced endoscopic techniques are crucial to provide the greatest survival benefit for patients. ERCP with brushing, scraping, and intraductal biopsies is the preferred method of invasive imaging for biliary strictures requiring biliary drainage. EUS–FNA is favored when non-invasive imaging demonstrates a mass lesion associated with a stricture or when ERCP is unsuccessful in revealing a diagnosis. Cholangioscopy provides direct visualization of the biliary stricture and permits targeted tissue biopsy. Tissue samples taken from these procedures are undergoing experimental evaluation and processing to enhance diagnostic yield. The advances in tissue sampling and diagnosis may lead into a new era of personalized medicine by allowing for targeted gene therapy for MBS in the future. The role of confocal laser endomicroscopy continues to evolve given the high cost and provider variability associated with this technique. The recent advances described in this review have allowed for more comprehensive evaluation, understanding, and effective diagnosis of the disease process.

## Figures and Tables

**Table 1:** Sensitivity and specificity of various MBS diagnostic modalities.

Diagnostics of Malignant Biliary Strictures			
Modality	Sensitivity (%)	Specificity (%)	Reference
*Lab markers*			
CA 19-9	80	89–90	Hasan et al. [10]
CEA	30–68	75–95	Yue et al. [12]
*Non-invasive imaging*			
MRCP	38–90	70–85	Singh et al. [23]
CT	75–80	60–80	Singh et al. [23]
US	90–95	30–70	Kapoor et al. [6]
*Invasive Imaging*			
PTHC	71	48	Kim et al. [62]
ERCP			
Brushing cytology	23–56	95	Singh et al. [23]
Fluoroscopic biopsy	33–65	95	Singh et al. [23]
Brushing + fluoroscopic biopsy	60–70	95	Singh et al. [23]
Brushing + bile fluid	84	95	Roth et al. [27]
Brushing + miRNA	54–85	95	Le et al. [29]
Brushing + FISH	50–60	95	Kushnir et al. [31]
Brushing + MP	56	95	Kushnir et al. [31]
Brushing + FISH + MP	66–69	95	Kushnir et al. [31]
EUS–FNA	80	97	Nakai et al. [26]
Cholangioscopy	88.9	97.6	Kulpatcharapong et al. [34]
Cholangioscopy directed biopsy	71–100	96.7–100	Ayoub et al. [33]
